# Mitral regurgitation as a phenotypic manifestation of nonphotosensitive trichothiodystrophy due to a splice variant in *MPLKIP*

**DOI:** 10.1186/s12881-016-0275-5

**Published:** 2016-02-16

**Authors:** Khadim Shah, Raja Hussain Ali, Muhammad Ansar, Kwanghyuk Lee, Muhammad Salman Chishti, Izoduwa Abbe, Biao Li, University of Washington Center for Mendelian Genomics, Joshua D. Smith, Deborah A. Nickerson, Jay Shendure, Paul J. Coucke, Wouter Steyaert, Michael J. Bamshad, Regie Lyn P. Santos-Cortez, Suzanne M. Leal, Wasim Ahmad

**Affiliations:** Department of Biochemistry, Faculty of Biological Sciences, Quaid-i-Azam University, Islamabad, 45320 Pakistan; Center for Medical Genetics, Ghent University Hospital, 9000 Ghent, Belgium; Center for Statistical Genetics, Department of Molecular and Human Genetics, Baylor College of Medicine, Houston, TX 77030 USA; Department of Biochemistry, Hazara University, Mansehra, Khyber Pakhtunkhwa 21300 Pakistan; Department of Genome Sciences, University of Washington, Seattle, Washington 98195 USA

**Keywords:** Autosomal recessive, Cardiomyopathy, Mitral regurgitation, *MPLKIP*, Nonphotosensitive, Phenotypic expansion, Splice mutation, Trichothiodystrophy

## Abstract

**Background:**

Nonphotosensitive trichothiodystrophy (TTDN) is a rare autosomal recessive disorder of neuroectodermal origin. The condition is marked by hair abnormalities, intellectual impairment, nail dystrophies and susceptibility to infections but with no UV sensitivity.

**Methods:**

We identified three consanguineous Pakistani families with varied TTDN features and used homozygosity mapping, linkage analysis, and Sanger and exome sequencing in order to identify pathogenic variants. Haplotype analysis was performed and haplotype age estimated. A splicing assay was used to validate the effect of the *MPLKIP* splice variant on expression.

**Results:**

Affected individuals from all families exhibit several TTDN features along with a heart-specific feature, i.e. mitral regurgitation. Exome sequencing in the probands from families ED168 and ED241 identified a homozygous splice mutation c.339 + 1G > A within *MPLKIP*. The same splice variant co-segregates with TTDN in a third family ED210. The *MPLKIP* splice variant was not found in public databases, e.g. the Exome Aggregation Consortium, and in unrelated Pakistani controls. Functional analysis of the splice variant confirmed intron retention, which leads to protein truncation and loss of a phosphorylation site. Haplotype analysis identified a 585.1-kb haplotype which includes the *MPLKIP* variant, supporting the existence of a founder haplotype that is estimated to be 25,900 years old.

**Conclusion:**

This study extends the allelic and phenotypic spectra of *MPLKIP-*related TTDN, to include a splice variant that causes cardiomyopathy as part of the TTDN phenotype.

## Background

Trichothiodystrophy (TTD) is an autosomal recessive disorder characterized by dry and easily broken brittle hair. Hair lacks sulfur, an element that normally gives hair its strength. The signs and symptoms of TTD vary widely; mild cases may involve only hair and in more severe cases additional features include intellectual disability, dwarfism, microcephaly, abnormal facial features, premature aging, ichthyosis, nail dystrophies, infertility and proneness to respiratory infections [1]. TTD is divided into two forms i.e. photosensitive (TTD1-3) and nonphotosensitive (TTD4 or TTDN1; MIM 234050) based on the presence or absence of clinical and cellular photosensitivity, respectively.

Majority of the photosensitive TTD patients have defects in nucleotide excision repair pathway genes. *ERCC2* (MIM 126340) variants cause TTD1 (MIM 601675) and are also responsible for cerebrooculofacioskeletal syndrome (MIM 610756) and xeroderma pigmentosum group D (XPD; MIM 278730) [2]. Variants in *ERCC3* (MIM 133510) cause not only TTD2 (MIM 616390) but also xeroderma pigmentosum complementation group B (XPB; MIM 610651) [3]. Autosomal recessive variants in *GTF2H5* (MIM 608780) underlie TTD3 (MIM 616395) [4]. These genes encode different subunits of the general transcription factor IIH (TFIIH), which is also involved in global genome repair and transcription-coupled repair. For nonphotosensitive TTDN only causal mutations in the *MPLKIP* (MIM 609188) gene have been reported [5]. *MPLKIP* consists of two coding exons on chromosome 7p14.1 that encode nucleus-restricted Plk1-interacting protein, and has ubiquitous expression in brain, heart, lung, placenta, epidermis and hair follicles, among others [5, 6]. The precise function of MPLKIP has not yet been elucidated but its nuclear localization signifies its role as a transcriptional regulator of genes relevant for metabolic pathways that are central to the outcome of TTDN1 [5].

In the present study, we identified three consanguineous Pakistani families displaying features of TTDN. Linkage analysis and homozygosity mapping coupled with exome sequencing identified a splice site variant c.339 + 1G > A in *MPLKIP* that segregates with TTDN in three families. The expression of MPLKIP in constructs carrying the splice variant in HEK293 cells confirmed unusual splicing, which results in an abnormal protein product. Additionally mitral regurgitation was identified in all affected individuals, expanding the TTDN1 spectrum caused by *MPLKIP* variants.

## Methods

### Recruitment of human subjects and clinical examination

**Fig. 1:**
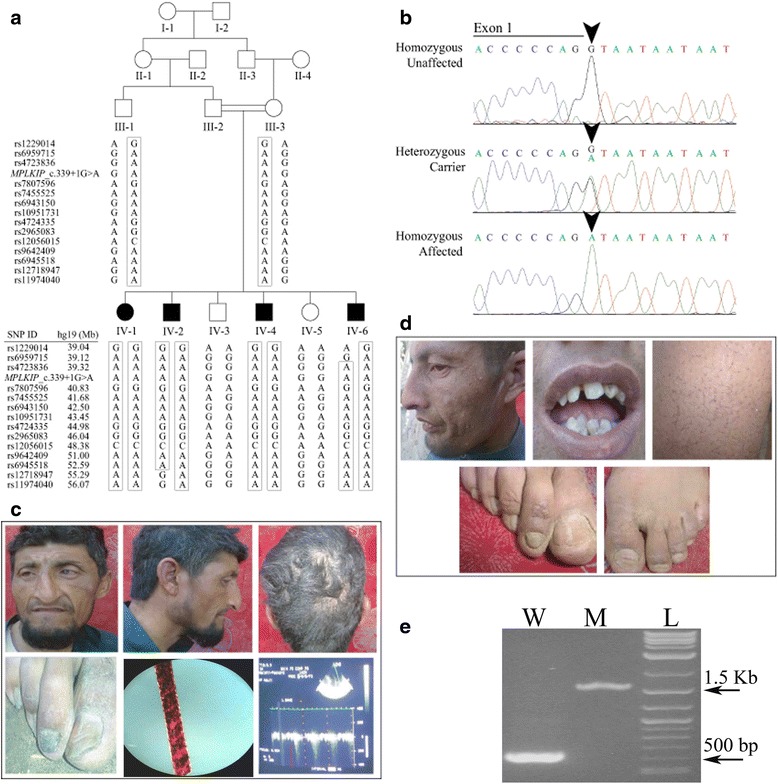
**a** Pedigree of consanguineous Pakistani family ED168 showing co-segregation of TTDN with the SNP haplotype including the *MPLKIP* c.339 + 1G > A variant. A DNA sample from the proband IV-1 was submitted for exome sequencing. The left panel lists the SNP markers and corresponding hg19 physical positions on chromosome 7. **b** Chromatograms showing the splice variant as homozygous in an affected individual and heterozygous or wild type in unaffected individuals. **c** Affected individual IV-2 (35 years old) has cataract and corneal bulging on the left eye, absent eyelashes, sparse eyebrows, patchy hair loss on scalp and toenail dystrophy with grooved ridges. Scalp hair examination with light microscopy shows characteristic tiger tail pattern. Echocardiogram from individual IV-2 revealed extra downward peaks, suggestive of mitral regurgitation. **d** Affected individual IV-4 (27 years old) has sparse eyebrows, eyelashes and facial and leg hair. He also has irregular teeth with hypodontia and dystrophic nails with grooved ridges. **e** cDNA analyses of the wild type transcript (lane W, 585 bp) and mutated MPLKIP transcript (lane M, 1569 bp) expressed in HEK293 cells, as compared to lane L showing the 2-Log DNA Ladder. The position of 500 bp and 1500 bp products in ladder is marked with arrows

**Fig. 2:**
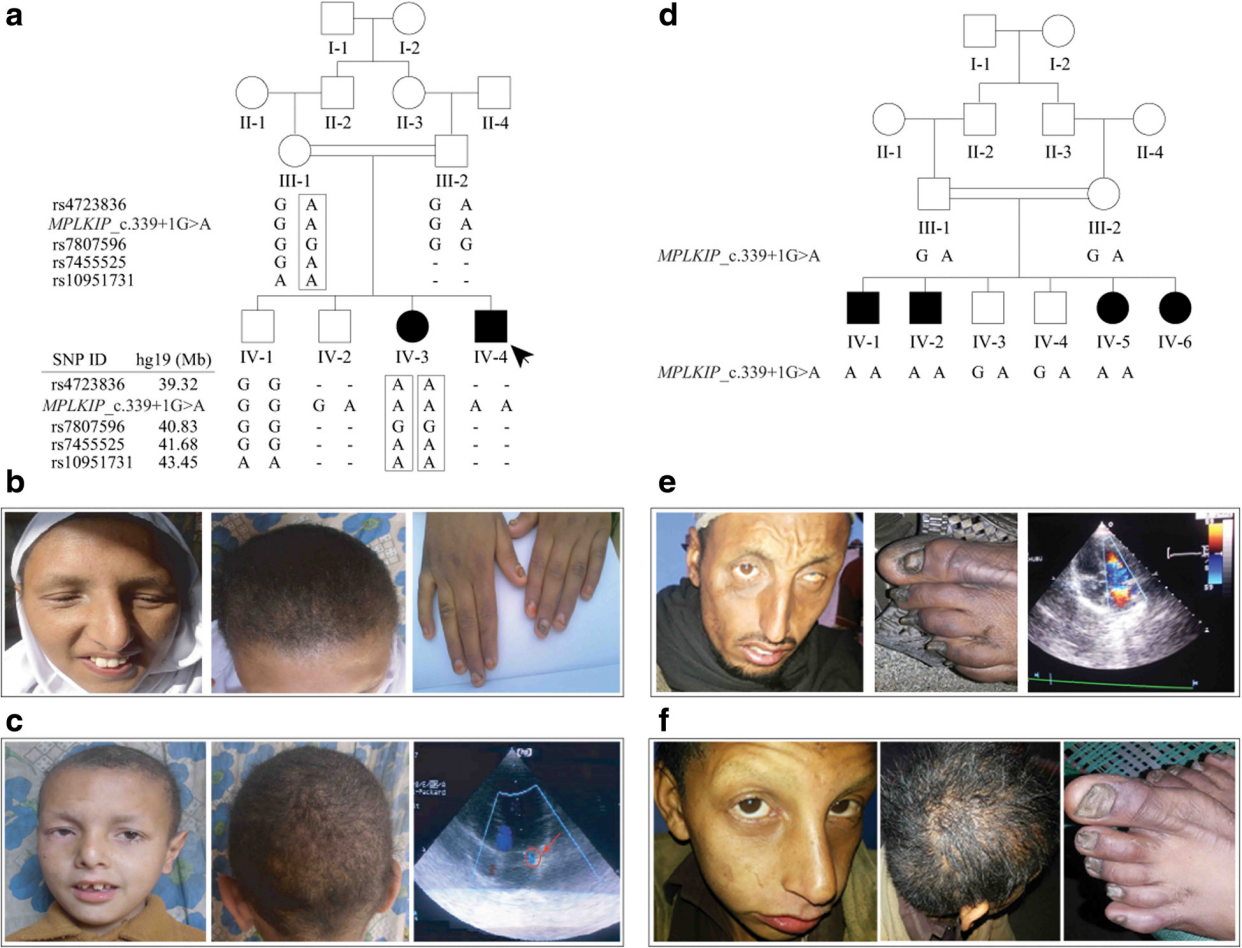
**a** Pedigree of a consanguineous Pakistani family ED210 showing co-segregation of TTDN with the 4.13-Mb haplotype that includes the *MPLKIP* c.339 + 1G > A variant and four SNPs. The haplotype in family ED210 is included within the haplotype from family ED168. **b** Affected individual IV-3 (17 years old) has sparse eyebrows and eyelashes, crooked beaked nose, flat malar eminences, retrognathia, patchy hair loss on scalp, and nail dystrophy. **c** Aside from hair abnormalities, the proband IV-4 (8 years old) also has hypodontia and irregular teeth. The echocardiogram from IV-4 shows mitral regurgitation. **d** Pedigree of third family, ED241 showing co-segregation of TTDN with the *MPLKIP* c.339 + 1G > A variant. The affected individuals IV-1 (**e**) and IV-2 (**f**) show sparse to absent eyebrows and eyelashes, brittle and sparse hairs on scalp and dystrophic toenails. The echocardiogram from IV-1 (**e**) also shows mitral regurgitation

Approval of the study was obtained from the Institutional Review Boards of: Quaid-i-Azam University, Islamabad, Pakistan; Baylor College of Medicine and Affiliated Hospitals, Houston, Texas, USA; and Center for Medical Genetics, Ghent University Hospital, Ghent, Belgium. Written informed consent was obtained from the patient and all family members for publication of genetic data, clinical findings and any accompanying images. Copies of the written consent were made available to this journal’s Editor for review. The three families ED168, ED210 and ED241 were recruited from rural areas of Khyber Pakhtunkhwa province, Pakistan. The pedigree structures (Figs. 1a, 2a and d) provided convincing evidence of an autosomal recessive mode of inheritance of TTDN. Affected members of these families underwent a complete physical examination and 2-D echocardiogram at a local government hospital.

### DNA extraction and genotyping

DNA was extracted from venous blood samples collected from four affected and four unaffected members of family ED168 (Fig. 1a), two affected and four unaffected members of family ED210 (Fig. 2a) and three affected and four unaffected members of family ED241 (Fig. 2d). Eight DNA samples from family ED168 were submitted for whole-genome scan using the Infinium® HumanCoreExome Chip (Illumina, USA), which consists of >500,000 SNP markers. Homozygosity mapping and linkage analysis were performed using HomozygosityMapper and MERLIN, respectively [7, 8]. Multipoint linkage analysis was performed using a disease allele frequency of 0.001 and an autosomal recessive mode of inheritance with complete penetrance. Allele frequencies for SNP markers were estimated using founders and reconstructed founders from 16 Pakistani families genotyped using the same array. The genetic map positions were based on the Rutgers combined linkage-physical map (NCBI, GRCh37/hg19) [9].

### Exome and Sanger sequencing

Exome sequencing was carried out using two DNA samples, the affected female IV-1 from family ED168 (Fig. 1a) and the affected male individual IV-2 from family ED241 (Fig. 2d). For ED168 IV-1, sequence capture was performed in solution with the Roche NimbleGen SeqCap EZ Human Exome Library v2.0 to target approximately 36.6 Mb of coding region. Subsequently the captured regions were sequenced on an Illumina HiSeq. FASTQ files were aligned to the human reference sequence (hg19) by using Burrows-Wheeler Aligner [10]. The Genome Analysis Toolkit (GATK) was used for realignment of regions containing indels, recalibration of base qualities, and variant detection and calling [11]. The variant sites were annotated using SeattleSeq137.

For ED241 IV-2, exome capture was carried out using the SureSelectXT Human All Exon V5 Enrichment Kit (Agilent, Santa Clara CA, USA) and sequencing was performed on a NextSeq 500 platform with paired-end 150-bp reads. The CLC Genomics Workbench v7.0.3 (CLC Bio, Qiagen, Aarhus, Denmark) software was used for read mapping against the human genome reference sequence (hg19), duplicate read removal, coverage analysis, and variant calling. Variants were annotated with an in-house software package, which makes use of the Ensembl API and Alamut Batch. To reduce the number of variants, synonymous variants with no predicted splice site disruption, variants with an allelic frequency in the global 1000 Genomes population >10 % and technical variants (i.e. coverage <3 and observation in only one sequence direction) were filtered out. The possible impact of variants on the protein level and on the correct splicing was further assessed by Alamut [12], PolyPhen-2 [13], and SIFT [14].

The exome sequence data for families ED168 and ED241 were independently analyzed focusing on the linkage region for family ED168. Occurrence of homozygous variants was checked in public databases [dbSNP, 1000 Genomes, Exome Variant Server (EVS), Exome Aggregation Consortium (ExAC)] and exome sequence data of 218 unrelated Pakistani individuals who have various Mendelian traits but not TTDN. Functional annotation was performed using the Combined Annotation Dependent Depletion (CADD) [15] and dbNSFP [16].

Sanger sequencing was performed to confirm co-segregation of a homozygous splice site variant in intron 1 of *MPLKIP* (NM_138701.3) with TTDN using eight and seven DNA samples from families ED168 and ED241, respectively, and primers 5^/^ GTACGGCTCTGCCACTCTTT 3^/^ and 5^/^ CGTACGGGAGCAGTCACTC 3^/^. When family ED210 was identified, all DNA samples that are available from family members were sequenced for the two coding exons of *MPLKIP.* In four individuals from family ED210 (Fig. 2a), additional SNP markers close to the *MPLKIP* splice variant were Sanger-sequenced in order to verify the occurrence of a founder haplotype. To determine the presence of the *MPLKIP* splice variant within the general population, DNA samples from 142 Pakistani control individuals were also sequenced.

### Haplotype analysis

Genotypes for the *MPLKIP* variant and 14 SNPs within a 585.1-kb haplotype which are homozygous in the two exomes from ED168 and ED241 were examined in 218 in-house exomes from unrelated Pakistani individuals, all of whom do not have TTDN but 48 of whom have partially overlapping phenotypes, i.e. intellectual disability, corneal disease, nail dystrophy, and hair loss. The DMLE+ program was used to estimate the age of the haplotype [17]. For haplotype age estimation, the following parameters were used: autosomal recessive model of inheritance; Pakistani population growth rate of 1.6 %, with an overall population of ~186,000,000; proportion of TTDN phenotype as 1 per 1 million; proportion of the population sampled at 7.5×10^-8^; 25-year intervals for each generation; and interpolated map positions in cM using the Rutgers combined linkage-physical map. A total of 1,000,000 burn-in iterations and 1,000,000 Markov chain Monte Carlo iterations were run simultaneously, and mean and 95 % confidence intervals (CI) for haplotype age based on number of generations were calculated using the last 10,000 iterations on each run.

### Splicing assay

The *MPLKIP* gene was amplified using phusion high fidelity DNA polymerase (New England Biolabs, MA, USA) using the genomic DNA of a single affected (IV-1) and an unaffected individual (IV-3) of family ED241, with forward and reverse primers carrying the restriction sites for Not1 (5′ TGAGCTAGCATGCAGCGACAGAATTTTCGG 3′) and Nhe1 (5′ TGAGCGGCCGCTGTTCCTGACACATGAAGCTTCC 3′) respectively at 5′ tails. The mutant and wild type amplicons were purified using the PCR purification kit (QIAGEN, Hilden, Germany) and subcloned in the topo vector using Zero blunt topo PCR Cloning Kit (Invitrogen life sciences, Darmstadt, Germany) and transformed into the One shot top10F* chemically competent cells (Invitrogen life sciences, Darmstadt, Germany), preceded by overnight incubation at 37 °C after plating on kanamycin-containing media. Next day the colonies containing the clone of interest (verified by sequencing) were processed for plasmid extraction using the QIAGEN mini prep kit. Double digestion was done on 5 μg of extracted plasmids and 2 μg of PCDNA3.1 by taking 15 units of NOT1 and NHE1 in buffer 2.1 (New England Biolabs, MA, USA) with a total volume of 100 μl at 37 °C for 2 h. The double-digest products were gel-purified using the QIAGEN QIAquick Gel Extraction Kit and processed for overnight ligation reaction at 16 °C using a 1:4 ratio of PCDNA3.1 and the mutant and wild type DNA in separate reactions with a total volume of 10 μl using T4 DNA ligase (Promega, Madison, USA). The ligated PCDNA3.1-mutant *MPLKIP* and PCDNA3.1-wild type *MPLKIP* were transformed again in One shot top10F* chemically competent cells, then incubated overnight at 37 °C after plating on ampicillin-containing media. The colonies were sequenced to verify the presence of the sequence of interest, followed by plasmid extraction using the QIAGEN miniprep kit. Prior to transfection, the HEK293 cells were seeded in a 12-well plate to make them at least 70 % confluent, following 24 h of incubation at 37 °C in 5%CO_2_. Using standard protocol 2 μg of extracted plasmids were introduced for transfection using fugene HD transfection reagent (Promega, Madison, USA). The transfection of mutant and wild type *MPLKIP* constructs in HEK293 cells was done in triplicate. Following 48 h of incubation at 37 °C in 5%CO2, with change of media after the initial 24 h, the cells were processed for RNA extraction using the QIAGEN Rneasy Minikit. cDNA was synthesized using 1 μg of extracted RNA random hexamer primers and M-MuLVreverse transcriptase (New England Biolabs, MA, USA). The cDNA was amplified using the *MPLKIP*-specific primer set used for first amplification. PCR products were analyzed on 1 % agarose gel stained with ethidium bromide. The amplified products were purified using the QIAGEN QIAquick Gel Extraction Kit and sequenced bidirectionally.

## Results

All affected individuals of three families were born full-term. The affected individuals presented with similar clinical features of TTDN (Figs. 1, 2, Table 1), including: hair abnormalities such as brittle hair, slow-growing sparse scalp hair with patchy loss, sparse eyebrows, eyelashes and body hair; aged appearance; recurrent rhinosinusitis; and mild intellectual disability with slurred speech. Affected adults were within the normal range for stature, while two children ED241 IV-5 (12 year-old female) and ED210 IV-4 (8 year-old male; Table 1) were below 1 and 2 standard deviations, respectively, from the mean age-based height of Pakistani children [18]. X-rays were not available to determine bone age for these two children. Polarized light microscopy of hair shafts from affected individual IV-2 of family ED168 showed irregular surfaces and fractures through the hair shafts and the characteristic tiger tail pattern (Fig. 1c), which are suggestive of low sulfur content. None of the affected individuals complained of UV sensitivity or ichthyosis. Common facial characteristics among affected individuals from these families include beaked noses with crooked nasal bridges and seemingly flat malar regions with either mandibular retrognathia or prognathia (Figs. 1, 2). Additional features affecting nails, teeth and eyes were identified in some but not all affected family members (Table 1). Eight of nine affected individuals were confirmed to have a high-arched palate, while only individual IV-2 from family ED168 had a history of epilepsy and corneal opacity upon ophthalmologic examination (Table 1, Fig. 1c). On cardiac examination, all affected individuals were found to have mitral regurgitation, while the cardiac examination of twelve unaffected individuals of three families (Figs. 1, 2) was unremarkable for any cardiac abnormality including mitral regurgitation. Echocardiography performed on individuals IV-2 of family ED168 (Fig. 1c), IV-4 of family ED210 (Fig. 2c) and IV-2 of family ED241 (Fig. 2e) confirmed the diagnosis of mitral regurgitation in these affected individuals.

**Table 1:** Clinical features of individuals with nonphotosensitive trichothiodystrophy

Individuals	ED168				ED210		ED241		
	IV-1	IV-2	IV-4	IV-6	IV-3	IV-4	IV-1	IV-2	IV-5
Gender	Female	Male	Male	Male	Female	Male	Male	Male	Female
Age (years)	38	35	27	23	17	8	25	15	12
Height	5′4″	5′8″	5′6″	5′6″	4′9″	3′4″^a^	5′7″	5′1″	4′5″^a^
Gestation	Normal	Normal	Normal	Normal	Normal	Normal	Normal	Normal	Normal
Hair features									
-Brittle hair	Yes	Yes	Yes	Yes	Yes	Yes	Yes	Yes	Yes
-Dystrophic shaft	Yes	Yes	Yes	Yes	Yes	Yes	Yes	Yes	Yes
-Hair growth	Slow	Slow	Slow	Slow	Slow	Slow	Slow	Slow	Slow
-Diffuse hair fall	Yes	Yes	Yes	Yes	Yes	Yes	Yes	Yes	Yes
Eyebrows	Sparse	Sparse	Sparse	Sparse	Sparse	Sparse	Sparse	Absent	Absent
Eyelashes	Sparse	Sparse	Sparse	Sparse	Sparse	Sparse	Sparse	Absent	Absent
Beard	-	Less dense	Less dense	Less dense	-	-	Less dense	-	-
Aged appearance	Yes	Yes	Yes	Yes	Yes	Yes	Yes	Yes	Yes
Intellectual disability	Mild	Mild	Mild	Mild	Mild	Mild	Mild	Mild	Mild
Speech	Slurred	Slurred	Slurred	Slurred	Slurred	Slurred	Slurred	Slurred	Slurred
Epilepsy	No	Yes	No	No	No	No	No	No	No
Ocular features									
-Blurred vision	Yes	Yes	No	No	Yes	Yes	Yes	Yes	Yes
-Corneal bulging	Yes	Yes	No	No	No	No	No	No	No
-Corneal opacity	No	Yes	No	No	No	No	No	No	No
Recurrent rhinosinusitis	Severe	Severe	Mild	Mild	Mild	Mild	Mild	Mild	Mild
High-arched Palate	Yes	?	Yes	Yes	Yes	Yes	Yes	Yes	Yes
Teeth	Normal	Normal	Irregular, Hypodontia	Normal	Normal	Irregular, Hypodontia	Irregular	Normal	Normal
Skeletal structure	Normal	Normal	Normal	Normal	Normal	Normal	Normal	Normal	Normal
Ichthyosis	No	No	No	No	No	No	No	No	No
UV sensitivity	No	No	No	No	No	No	No	No	No
Nails	Normal	Grooved	Grooved	Normal	Thick	Normal	Dystrophic	Dystrophic	Dystrophic
Mitral regurgitation	Yes	Yes	Yes	Yes	Yes	Yes	Yes	Yes	Yes

^a^Height for these two children fall below 1–2 standard deviations from the mean for Pakistani children of similar age

Using genome-wide genotypes from family ED168, homozygosity mapping and linkage analysis identified a 16.17-Mb homozygous region between SNP markers rs6959715 and rs12718947 on chromosome 7p14 (Fig. 1a), with maximum multipoint LOD score of 3.26. From the exome sequence data of ED168 IV-1, 63 homozygous variants were found within the homozygous region. Among the called variants, only one coding variant was not reported in public databases, namely splice variant c.339 + 1G > A in the *MPLKIP* gene. Similarly from the exome sequence data of ED241 IV-2, the homozygous *MPLKIP* splice variant was identified as the strongest candidate among the observed variants. On the other hand, the *MPLKIP* c.339 + 1G > A variant was not found in 218 in-house exomes from unrelated non-TTDN Pakistani individuals or in Sanger sequence data from 284 Pakistani control chromosomes.

The *MPLKIP* splice variant has a scaled CADD score of 18.51, denoting that the variant is highly likely to be deleterious. Functional analysis of the *MPLKIP* splice variant c.339 + 1G > A confirmed pathogenicity at level of transcription, with abrupt splicing of two exons due to loss of the canonical splice donor site, consequently resulting in retention of the intervening single intron of 969 bp (Fig. 1e). The resultant transcript featured with a frameshift likely to result in formation of a truncated MPLKIP protein of 131 amino acids as compared to its wild type counterpart with 179 amino acids. Sanger sequencing of the splice variant in all available samples from both families, ED168 and ED241, confirmed co-segregation with the TTDN phenotype (Figs. 1a and b, 2a and b). Family ED210 was ascertained to have similar TTDN features as family ED168, thus the two coding exons of *MPLKIP* were sequenced using six DNA samples from family ED210. The same *MPLKIP* c.339 + 1G > A variant was found to co-segregate with TTDN in family ED210 (Fig. 2a).

Comparison of four SNP genotypes surrounding the *MPLKIP* gene revealed that affected individuals from families ED168 and ED210 share a 4.13-Mb haplotype from rs4723836 to rs10951731 (Fig. 2a), implicating the splice variant might arise from a common founder. Closer examination of the exome variants surrounding the *MPLKIP* variant identified 14 SNPs that are homozygous in both exomes from affected individuals ED168 IV-1 and ED241 IV-2 (Table 2). Using genotypes from 218 unrelated Pakistani individuals and the DMLE+ software, the 585.1-kb haplotype was estimated to be at least 25,900 years old [95 % CI: 25,350-26,375].

**Table 2:** Haplotype including homozygous exome variants from TTDN families ED168 and ED241

hg19 Position	Reference Allele	Alternate Allele	ExAC South Asian MAF	Gene	Variant	Type	#Heterozygous^a^	#Homozygous^a^
39606107	G	A	0.10	*YAE1D1*	c.90G > A	synonymous	41	3
39608877	T	C	0	*YAE1D1*	c.130 -1228 T > C	intronic	0	0
39609087	C	G	0	*YAE1D1*	c.130 -1018C > G	intronic	0	0
39609442	A	T	0	*YAE1D1*	c.130 -663A > T	intronic	0	0
39610177	A	G	0.10	*YAE1D1*	c.202A > G^b^	missense	41	3
39610241	C	G	0.11	*YAE1D1*	c.251 + 15C > G	intronic	13	0
39611748	A	G	0	*YAE1D1*	c.252 -128A > G	intronic	0	0
39611819	G	A	0	*YAE1D1*	c.252 -57G > A	intronic	0	0
39856354	T	C	0	*NA*	NA	intergenic	1	0
39874259	G	A	0	*NA*	NA	intergenic	28	11
39874281	T	C	0	*NA*	NA	intergenic	28	11
40087752	A	G	0	*CDK13*	c.2600 + 276A > G	intronic	0	3^c^
40117364	A	G	0	*CDK13*	c.2781 -240A > G	intronic	1	0
40173827	C	T	0	*MPLKIP*	c.339 + 1G > A	splice	0	0
40191226	C	G	0	*C7orf10*	c.121 + 16507C > G	intronic	1	0

^a^Based on 218 in-house exomes from unrelated Pakistani individuals with non-TTDN phenotypes

^b^This *YAE1D1* p.(Lys68Glu) variant is predicted to be benign by 9 out of 9 bioinformatics prediction tools from dbNSFP

^c^The 3 exomes that are homozygous for this variant are from individuals with nonsyndromic hearing impairment

NA, not applicable

## Discussion

In this report we present three unrelated Pakistani families with an *MPLKIP* splice variant co-segregating with TTDN. To date at least 13 families and probands with TTDN were identified to have homozygous or compound heterozygous *MPLKIP* mutations. Of the identified mutations, only one missense variant has been identified in an Amish family [5], while truncating deletions were found in individuals of European, Middle Eastern or Moroccan descent [19, 20]. The TTDN features identified in the three Pakistani families reported here overlapped with clinical findings in *MPLKIP* variant carriers with TTDN, particularly for hair abnormalities, intellectual disability and susceptibility to infections. In the Pakistani families, variable features such as nail dysplasia, tooth irregularities, facial characteristics, ocular disorders and epilepsy were identified as well.

Notably all affected individuals from the Pakistani families have mitral regurgitation which is probably due to cardiomyopathy, a feature that has not been reported as part of the TTDN spectrum specifically caused by *MPLKIP* mutations. In an earlier report, two Swiss sisters with TTDN were documented to have dilated cardiomyopathy with moderate mitral regurgitation [21], while a report on two infants who died early due to TTDN described pulmonic stenosis in both cases [22]. However we have no means to verify whether these TTDN patients also carry *MPLKIP* mutations. In a review of clinical findings in TTD/N patients, murmurs or septal defects were reported but some patients clearly have TTD rather than TTDN, while in other reports the presence or absence of photosensitivity was not documented [1]. In other cases, the heart defect was ruled out at autopsy or deemed coincidental e.g. murmur due to sideroblastic anemia, or isolated congenital heart defects which have a much higher incidence than TTD or TTDN [1, 21, 23]. For families ED168 and ED241, across the exome the *MPLKIP* splice mutation is the only rare homozygous variant that is predicted to be deleterious or damaging and this variant segregates with both TTDN and mitral regurgitation, supporting the inclusion of cardiomyopathy as a feature of TTDN due to *MPLKIP* variants. Additionally the variant occurs within a small founder haplotype that is shared by the two families, which further strengthens the evidence that the same founder variant is responsible for the rare TTDN phenotype including the cardiomyopathy.

MPLKIP binds to serine/threonine kinase PLK1, which plays critical roles in multiple cell cycle events, including mitotic entry, centrosome maturation and separation, spindle pole integrity, kinetochore attachment, and cytokinesis [24, 25]. MPLKIP colocalizes with PLK1 during mitosis, and overexpression or inhibition of MPLKIP results in nuclear fragmentation or disrupted mitotic spindles, suggesting the importance of PLK1 regulation by MPLKIP in cell cycle maintenance [26]. Phosphorylation of MPLKIP by CDK1 at multiple serine or threonine residues is required for interaction of MPLKIP with PLK1 [26]. While both MPLKIP and PLK1 are expressed in multiple tissues that are affected by TTDN including brain, lung, heart and skin [5, 6, 27], we did not identify TTDN phenotypes affecting organs where adult expression of MPLKIP is highest, such as in liver, kidney, skeletal muscle and pancreas [6]. On the other hand, MPLKIP is expressed in fetal skin and fibroblasts [5], while PLK1 expression is highest in tissues with actively proliferating cells (e.g. gonads, cancers) [27]. PLK1 is also expressed in neural progenitor cells and gradually diminishes during neurogenesis [28], but is increased in brain cells of Alzheimer disease patients [29]. Similarly in heart, expression of PLK1 mRNA and protein progressively decreases during development and disappears in the adult [30], but is upregulated in cardiomyocytes during regeneration post-injury [31]. The unusual splicing due to the *MPLKIP* splice variant as validated by the splice assay confirmed formation of a frameshift transcript due to a retained intron (Fig. 1e). The transcript with premature termination signal at codon 132 is predicted to result in the loss of a putative phosphorylation site at Ser133. The loss of the phosphorylation site is expected to underlie the TTDN pathogenesis observed in our families. Taken together this evidence suggests that non-functional MPLKIP due to protein truncation and non-phosphorylation might lead to failed interaction of MPLKIP with PLK1, which plays a role in development of various organs including skin, brain and heart.

## Conclusion

Overall our study expands the allelic and phenotypic spectra of *MPLKIP-*related TTDN, to include a splice variant that causes cardiomyopathy as part of the TTDN phenotype.

## Abbreviations

CADD: Combined Annotation Dependent Depletion
ExAC: Exome Aggregation Consortium
EVS: Exome Variant Server
GATK: Genome Analysis Toolkit
PLK: Polo-like kinase
TTD: (photosensitive) trichothiodystrophy
TTDN: Nonphotosensitive trichothiodystrophy
